# Identification of QTLs and Key Genes Enhancing Lodging Resistance in Soybean Through Chemical and Physical Trait Analysis

**DOI:** 10.3390/plants13243470

**Published:** 2024-12-11

**Authors:** Wanying Zhao, Depeng Zeng, Caitong Zhao, Dezhi Han, Shuo Li, Mingxing Wen, Xuefeng Liang, Xianfeng Zhang, Zhihua Liu, Shahid Ali, Zhenfeng Jiang

**Affiliations:** 1 Key Laboratory of Soybean Biology in Chinese Ministry of Education, College of Agriculture, Northeast Agricultural University, Harbin 150030, China; s220301053@126.com (W.Z.); zdp926498@163.com (D.Z.); m18846773398@163.com (C.Z.); lishuo19991030@163.com (S.L.); wenmingxing0302@163.com (M.W.); liangxf1230@163.com (X.L.); 2 Heihe Branch of Heilongjiang Academy of Agricultural Sciences, Heihe 164300, China; handezhi2008@163.com; 3 The Training Center of the Undergraduate, Northeast Agricultural University, Harbin 150030, China; 0451zhangxianfeng@163.com; 4 College of Resources and Environment, Northeast Agricultural University, Harbin 150030, China; zhihua-liu@neau.edu.cn; 5 Guangxi Key Laboratory of Agro-Environment and Agro-Products Safety, Key Laboratory of Crop Cultivation and Physiology, College of Agriculture, Guangxi University, Nanning 530004, China; shahidsafi926@gmail.com

**Keywords:** lodging, plant density, QTLs, soybean, temperature

## Abstract

Lodging of soybean (*Glycine max* (L.) Merril.) significantly reduces seed yield and quality, particularly in high-yielding environments. This phenomenon occurs when stems weaken under the weight of the plants, complicating harvesting. This study investigated the relationship between soybean stem chemical composition, physical traits, and lodging resistance to improve yield and resilience. We found that as plant density increased, stem hardness decreased, and the elasticity increased, heightening the risk of lodging. Conversely, high temperature (28 °C) boosted lignin, cellulose and pectin content in the stem cell walls, enhancing the lodging resistance. Additionally, after excluding differences in phylogenetic relationships through cluster analysis, we mapped environment-stable genes linked to lodging resistance and identified new QTLs on Chr3 and Chr16. Candidate genes associated with these QTLs were confirmed using qRT–PCR and hormone treatments across diverse soybean varieties. It was found that the expression of stem tip genes was closely related to stem node diameter. These findings provide a theoretical foundation for breeding high-yielding soybean varieties with improved lodging resistance, and advance efforts to develop resilient soybean cultivars.

## 1. Introduction

Lodging caused by high-density planting is a persistent and significant challenge in crop production. As the global population grows, increasing crop yields through high-density planting has become essential. However, excessive planting density can lead to severe lodging, which disrupts nutrient accumulation and transport [1] and consequently reduces yield [2]. Therefore, achieving lodging resistance to maintain high crop yields has garnered considerable attention from researchers worldwide.

Crop stems bear the weight from above-ground biomass and environmental factors, such as leaves, wind, and rain, which are ultimately transferred to the basal internode. Thus, the mechanical properties of the stem, particularly the basal internode, are crucial indicators of lodging resistance. For example, stem bending strength (SBS) is an important factor of crop stem strength [3] with higher SBS values indicating stronger lodging resistance [4]. SBS is positively correlated with stem thickness and lodging resistance but negatively correlated with the lodging index [5]. Additionally, lodging resistance often depends on both the plant’s center of gravity and the internodes’ mechanical strength, which supports the plant’s upright position [6]. Specifically, basal internode diameter has a positive correlation with breaking strength [7]. For example, increases in culm diameter and wall thickness in rice are associated with enhanced physical strength [8,9]. Similarly, in wheat, larger stem diameter and wall thickness improve lodging resistance by increasing stem strength [10,11]. In soybeans, greater stem bending resistance correlates with increased lodging resistance [12]. Thus, enhancing stem strength is essential for improving the lodging resistance of soybean plants. Some existing studies have focused on the effects of plant stem composition and structure on lodging resistance but lack molecular-level research.

The research findings indicate that the physical characteristics of stem tissue and the chemical components of the cell wall are important in determining the lodging resistance of crops [13,14]. Chemical elements that contribute to stem strength include lignin, cellulose, hemicellulose, and pectin [15]. Lignin has a significant effect on stem strength and makes up 20% of the crop plants’ dry matter [16]. Crop stems mechanical strength rises with increasing lignin content, but a lower lignin content increases the possibility of lodging [17,18,19]. Several key enzymes for lignin synthesis have been discovered. Among them, the increased content of Phenylalanine Ammonia–Lyase (PAL), Caffeic Acid O–Methyltransferase (COMT), 4–Coumarate (4CL), Cinnamyl Alcohol Dehydrogenase (CAD), Cinnamoyl–CoA Reductase (CCR) shows a higher lignin content and stronger lodging resistance [20,21]. Additionally, cellulose and hemicellulose also affect the mechanical strength of the stem. The higher the content of stem cellulose and hemicellulose, the higher the stem strength and stronger lodging resistance in rice variety, which can be planted in a higher density and yield more. In addition, the shade-tolerant soybean stems have higher accumulation of cellulose and related enzyme activity, which improves the breaking strength and lodging resistance of the basal internode [22]. The relationship between stem tissue composition and lodging resistance is intricate and not yet fully understood, warranting further investigation.

To develop high-yield, lodging-resistant soybean varieties using a marker assistant selection, it is essential to identify the genetic information of key chromosome segments and screen candidate genes related to lodging resistance. Bulk Segregation Analysis (BSA) has been widely used in the breeding of new crop varieties [23]. Recently, BSA sequence (BSA–seq) has been successfully applied for gene cloning in crops. Research on lodging resistant rapeseed using BSA–seq found that *BnaA01g01910D* as a potential candidate gene responsible for the dwarf and compact phenotype observed in 4942C–5 [24]. Interestingly, BSA coupled with RNA sequencing (BSR–seq) is a novel approach to identify differentially expressed genes in crop species and to identify contrasting markers between two pools [25,26]. For instance, BSR-seq-derived wheat powdery mildew resistance molecules showed resistance from a single dominant gene on the 5DS chromosome [27]. BSR–Seq analysis of the soft date kiwi fruit cold tolerance response showed that the tolerant groups’ soluble sucrose and β–amylase activities were stronger than those of the susceptible group [28]. All of the aforementioned findings imply that BSA–seq and RNA–seq can speed up the process of identifying the key genes responsible for important agronomic traits and make gene cloning easier. In addition, several studies have demonstrated that BSA–seq and BSR–seq can effectively identify genes in populations where polymorphic markers have not yet discovered. Still, it is uncommon to find soybean cultivars with enhanced lodging resistance bred using the BSA–seq. This experiment helps to produce novel soybean varieties that are resistant to lodging and performs in-depth investigation from this standpoint.

## 2. Materials and Methods

### 2.1. Plant Materials Collections and Storage

A total of 85 soybean varieties from a natural population were obtained from Northeast Agricultural University Soybean Research Institute (Supplementary Table S1). Leaf and stem samples were collected by following the protocol of Jiang et al. [29]. These samples were quickly frozen in liquid nitrogen and stored at −80 °C for further analysis.

### 2.2. Soybean Seeding Growing Conditions

To aid in germination, healthy soybean seeds were selected, and the seed hilum was faced downward. To replicate the temperature conditions of the flourishing growth stage (R1 or so) of soybean, the plastic box was placed in an incubator with daytime temperatures of 23 °C (cold temperature) and 28 °C (warm temperature) and overnight temperature of 18 °C. The daytime was 16 h, and the overnight was 8 h. The effective radiation intensity for photosynthetic process was 500 μmoL m^–2^s^−1^. Plant growth was monitored and observed during the cultivation period.

### 2.3. Field and Pot Soybean Planting and Sampling Surveys

Two densities were used to evaluate each variety in the field experiment: H30 (high density: 300,000 plants/hectare) and L20 (low density: 200,000 plants/hectare). Each soybean variety’s seeds were planted in three replicates with a row length of 10 m and a ridge spacing of 0.65 m. Three duplicates of each density were used in the potted experiments, which are similarly carried out at high and low densities. Six soybean seedlings were kept in a plastic pot in L20, and ten were kept in H30.

The soybean plants in the pots and those that reached the full maturity stage (R8) in the field were sampled and tagged at the n–node stage (Vn), the first grain stage (R5), and the full maturity stage (R8) [30]. At R5 stage, measurements were made of the main stem height, the diameter of the basal internode (15 cm from the cotyledon), the break force, and the resilience. At R8 stage, measurements were made of the main stem height and the diameter of the first internode at the base of the soybean varieties in both pot and field experiments. The breaking force and resilience of the soybean stems were precisely measured using a texture analyzer (TA. XT plus, SMS, Surrey, UK).

### 2.4. Hormone Treatment of Soybean Seedlings

A vernier caliper was used to measure the diameter of the basal internode at 9 days of age, and each treatment was repeated three times. To measure the diameter of the basal internode, prepare 500 mmol·L^−1^ IAA and GA solutions, together with their inhibitors (1–phenylpropanamide, 5–uniconazole, etc.). Then, the solution was applied to the basal internode, and the diameter of the basal internode was measured 24 h later.

### 2.5. Determination of Cell Wall Component Content

#### 2.5.1. Determination of Lignin and Cellulose Content

The epicotyls of 15 days old seedlings were selected for sampling. Following this, and an oven-drying process at 65 °C, the lignin content was determined in accordance with Foster C E instructions [31]. The cellulose content was then ascertained using the Foster C E technique [32].

#### 2.5.2. Determination of the Total Pectin Content

After adding 1.5 mL of 80% ethanol to 0.1 g of the central portion of seedling epicotyl stem tissue, it is soaked in water at 85 °C for 10 min. After cooling in running water, it is centrifuged at 8000 rpm and 25 °C for 10 min. The supernatant is discarded and the precipitate is kept. A total of 1 mL of 80% ethanol is added to the precipitate, the above operation is repeated; then, 1 mL of extraction solution is added to the precipitate, mixed well, placed in a 95 °C water bath for 60 min, 8000 rpm, centrifuged at 25 °C for 10 min, and the supernatant is taken for testing. A total of 105 μL of sample and 630 μL of concentrated sulfuric acid is added to the EP tube in sequence. At the same time, a blank tube is prepared as a control. A total of 105 μL of distilled water and 630 μL of concentrated sulfuric acid is added to the blank tube. It is wrapped tightly with a sealing film and placed it in a water bath at 85 °C for 15 min. The absorbance value is read with a microplate reader at 530 nm.

### 2.6. RNA Extraction

E.Z.N.A. Plant RNA Kit kit was applied for RNA extraction. The steps are as follows: First, grind or chop the plant samples and add lysis buffer to break the cell walls and release RNA. Then, add detergent and proteinase K, shake or incubate the samples, and centrifuge to remove cellular impurities. Transfer the supernatant to an RNA purification column and use a binding buffer to allow RNA to bind to the column material. Next, wash away impurities using a wash buffer, and elute the purified RNA with elution buffer. Finally, assess the RNA quality and concentration using a UV spectrophotometer to ensure it was suitable for downstream applications.

### 2.7. RT–qPCR Analysis of Candidate Genes

Six soybean varieties were selected from both high diameter and low diameter groups based on the phenotypic data of 85 materials. Extraction of shoot tip tissue from the ternate compound–leaf stage for RNA extraction using TRIzol Reagent (Invitrogen, 15596–026, Carlsbad, CA, USA) was performed. Reverse transcription of extracted RNA into cDNA using the Tianhe Real-time quantitative PCR (RT–qPCR) kit was performed using SYBR qPCR Mix (Vazyme, Q711, Vazyme biotech, Nanjing, China) on the Light Cycler 480 System (Roche, Roche Diagnostics, Basel, Switzerland). Actin 11 (*Glyma.18G290800*) was selected as the internal reference and the expression level of candidate genes were calculated. Candidate gene-specific RT–qPCR primer sequences were designed using Primer Premier 5.0 (https://www.premierbiosoft.com/primerdesign/) (Table 1).

### 2.8. QTL Analysis via Euclidean Distance (ED)

The Euclidean distance (ED) algorithm was a method that used sequencing data to identify significant differences between pools and evaluate regions associated with traits [33]. Theoretically, there were differences in target trait-related sites between the two groups constructed by BSA, whereas other sites tend to be consistent, and the ED value should tend to be zero. The formula of the ED method was as follows. The greater the ED value, the greater the difference between the two mixing pools. H was the base frequency in the high pool and L was the base frequency in the low pool. This study used the median plus three standard deviations as the correlation value to eliminate background interference. Regions above the threshold were selected based on the threshold (median + 3SD) as regions related to the trait. Graphpad Pism9.5 was used to draw ED diagrams.

### 2.9. Function Analysis of the Candidate Genes

Candidate genes were functionally annotated using information from the Arabidopsis database (www.Arabidopsis.org) and the soybean database (www.Soybase.org).

### 2.10. Genotypes of Soybean Varieties Using a Soybean SNP Array

We used the soybean SNP array Geno Baits Soy10K for genotyping soybean varieties using young, tender leaf samples. Genomic DNA was extracted using the CTAB method, and the quality and concentration of the DNA were assessed by agarose gel electrophoresis and a nucleic acid protein quantifier (Qubit, Thermofisher, Waltham, MA, USA). A DNA fragment library with a size of 200–300 bp was constructed, and paired-end 150 bp reads were sequenced on the DNBSEQ-T7RS platform. Variant calling was performed using the UnifiedGenotyper module in GATK (version 3.5) with parameters “-dcov 1,000,000-minIndelFrac 0.15-glm BOTH -l INFO”. Variant quality control was based on the following parameters: “MQ0 >= 4 && ((MQ0/(1.0* DP)) > 0.1)” and “DP < 5 || QD < 2”. Variants with at least 5× depth were considered valid genotypes, while those with lower depths were considered missing. A heterozygous genotype was called MAF ≥ 0.2 and at least 4 reads supported each allele. Marker density was calculated using a 500 kb window, and the gap size between adjacent markers on the same chromosome was determined. Variants were annotated based on ANOVA with 3 kb in both directions. Only SNP sites were retained, and samples with a missing rate > 30% were filtered out. Sites with a missing rate > 50% were also excluded. Finally, loci with a minor allele frequency (MAF) ≥ 20 and biallelic were retained for analysis.

## 3. Results

### 3.1. Impact of Planting Density on Phenotypic Traits Related to Lodging Resistance

Planting density significantly influences the phenotypic traits related to the lodging resistance in soybeans, affecting both plant height and basal internode diameter across different growth stages. This study measured these traits in four soybean varieties at varying densities from vegetative (Vn) to full maturity (R8) stages. At the low density, the plant height of Heike71 increased from 14.20 cm at the Vn stages to 50.10 cm at the R8 stages, while the basal internode diameter grew from 4.71 mm to 6.32 mm (Table 2). Similar trends were observed in other soybean varieties. Under high-density conditions, plant height and basal internode diameter reached ranges of 22.07–27.31 cm and 4.61–5.08 mm at the Vn stage, respectively, and increased to 59.5–80.4 cm and 4.71–6.16 mm by the R8 stages.

The results indicated that increased planting density raises plant height but reduced stem diameter across all stages (Table 2). Lodging resistance was further assessed by measuring basal internode hardness and resilience. At low density, Heike88 showed the highest hardness (11,475.91 N), and lowest resilience (54.21 N), whereas Heike71 had the lowest hardness (5226.28 N), and the highest resilience (57.50 N). A negative correlation was observed between hardness and resilience within the same variety, suggesting that greater stem of hardness was associated with reduced resilience. As stem maturity progressed, both hardness and resilience increased regardless of variety. However, a higher planting density from 20 plants/m^2^ to 30 plants/m^2^ consistently decreased hardness while increasing resilience in the four soybean varieties (Table 3).

Heihe43, a widely cultivated high-yield variety, demonstrated a larger basal internode diameter at the R5 and R8 stages than the other three varieties. Moderate changes in stem diameter, hardness, and resilience in Heihe43 at different planting densities helped mitigate lodging by balancing stem strength and flexibility. These findings provide insights into the optimizing plant density for improved lodging resistance in high-yield soybean varieties.

### 3.2. Temperature Effects on Soybean Cell Wall Composition for Environmental Adaptation

**Figure 1:**
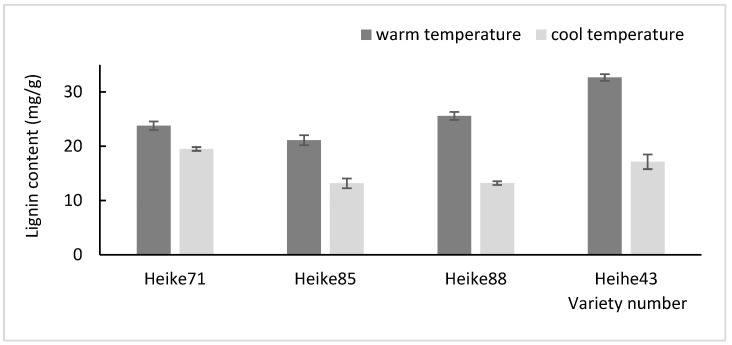
Determination of lignin content in soybean cell wall at high and low temperatures.

**Figure 2:**
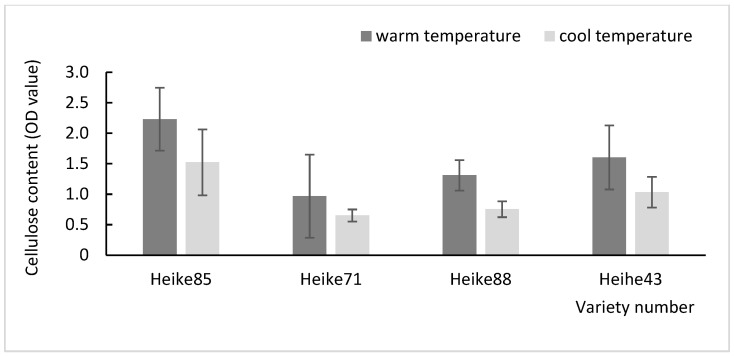
Determination of cellulose content in soybean cell walls at high and low temperatures.

**Figure 3:**
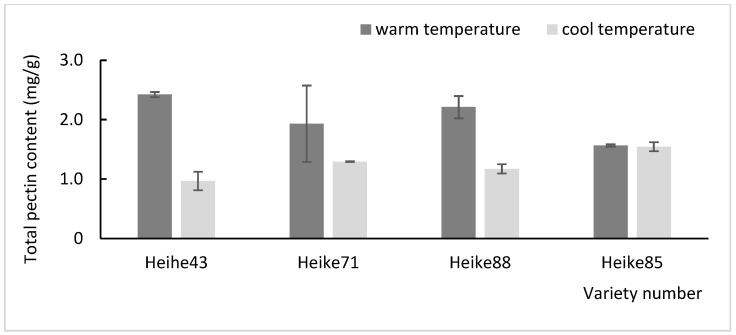
Determination of total pectin content of soybean cell wall at high and low temperatures.

The plant cell wall, unique to plants, is composed of various structural polysaccharides, including pectin, cellulose, hemicellulose, and lignin. These components accumulated in a temperature-dependent manner, with cellulose microfibrils providing a structural framework essential for plant growth and adaptation environmental fluctuation [15]. In this study, soybean seedlings were subjected to cool temperature (23 °C) at the seedling stage and warmer temperature (28 °C) at the flowering stage to assess the accumulation of lignin, cellulose, and pectin contents in the basal internode under different thermal conditions. The results demonstrated that the levels of these cell wall component were significantly lower at the cooler temperature compared to warmer temperature (Figure 1, Figure 2 and Figure 3). This suggests that, within certain limits, increasing the temperature promotes the accumulation of key cell wall constituents in soybean seedlings.

Further comparison among varieties revealed significant temperature-dependent differences in cell wall component content, especially in Heike88 and Heihe43. Both varieties exhibited a marked increase in lignin and pectin at higher temperature, with extremely significant differences (*p* < 0.01). These findings indicate that Heike88 and Heihe43 varieties are particularly responsive to environmental temperature changes, highlighting their potential adaptability through cell wall modifications in response to thermal variations.

### 3.3. Genetic Analysis and Phenotypic Variation of Lodging Resistance in Soybean Varieties

**Figure 4:**
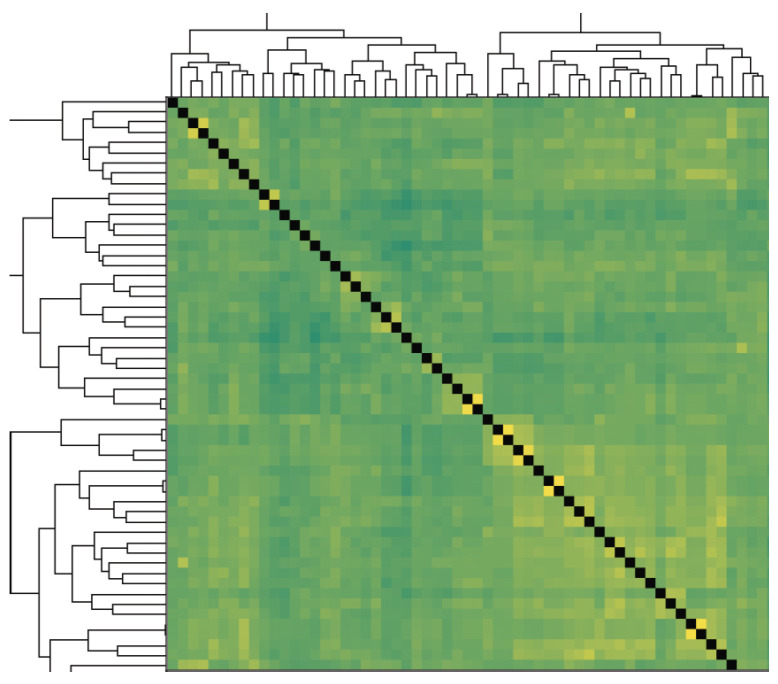
Genetic relationship clustering diagram of 85 soybean varieties. Note: Dark green in the figure indicates highly similar or closely related individuals or groups. Light green indicates moderate similarity or affinity. Yellow indicates weaker similarity or affinity.

This study evaluated the loading resistance of 85 soybean varieties by examining their genetic background and phenotypic traits. Due to the complex genetic structure of the natural population, a thorough genetic background analysis was performed to ensure the tested lines had similar ancestries. The analysis revealed that the primary ancestral parents of these varieties included Silihuang, Jinyuan, Baimei, and Keshan Silijia. Cluster analysis was conducted on 85 varieties, and it indicated that the test materials grouped into 2 distinct clusters, reflecting significant difference in basal internode diameters, suitable for Bulk Segregant Analysis (BSA) (Figure 4).

**Figure 5:**
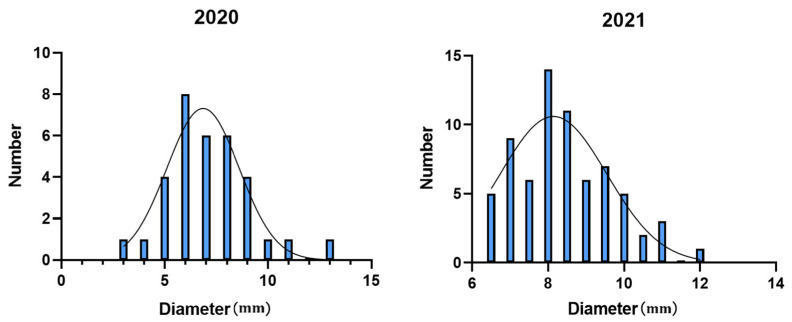
Frequency distribution of the first internode diameter of soybeans in natural soybean populations from 2020 to 2021.

Phenotypic assessment showed that the diameter of the basal internodes varied between years of studies; in 2020, they ranged from 2.57 to 12.95 mm (mean standard deviation: 1.91 mm), while in 2021, they ranged from 5.77 and 12.93 mm (mean standard deviation: 1.38 mm) (Figure 5). Statistical analysis indicated that the kurtosis and skewness values for diameter in both years were less than 1, (Table 4), suggesting that the population diameter data distribution approximated a normal distribution, making it suitable for QTL mapping.

### 3.4. Identification of Candidate Genomic Regions Associated with Target Traits in Soybean

**Figure 6:**
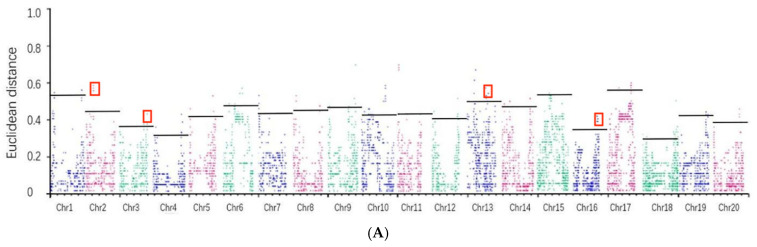

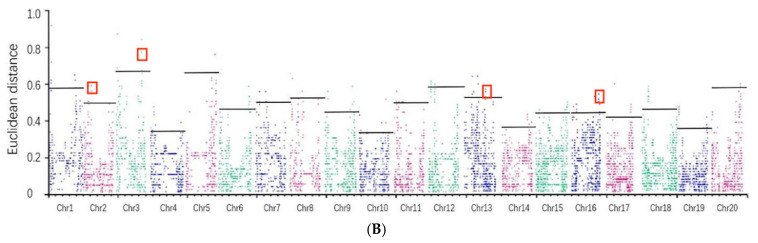
Distribution of the Euclidean distance (ED) correlation values on chromosomes. Note: the abscissa is the chromosome name. The colored points represent the ED value for each single nucleotide polymorphism (SNP) site. The red marks are SNP sites where the two ED graphs overlap. The gray line represents the threshold value for each point. (**A**) Analysis of the correlation values of the ED in 2021. (**B**) Analysis of the correlation values of ED in 2020.

To address background noise in genetic association studies, Hill et al. (2013) [33] proposed enhancing distance measurement by raising them to a reasonable power, specifically using “ED^x^”. In this study, we employed ED^2^ as the associated value and utilized the DISTANCE method to fit the effective distance (ED) values. Candidate genomic regions were identified based on the peak regions, with a significant threshold set at the median + 3 and standard deviations (SD) (Table 5). The analysis revealed that at a confidence level of 0.99, all chromosomes exceeded the defined threshold (Figure 6).

A comprehensive analysis combining results from two years identified four chromosomes (Chr_02, Chr_03, Chr_13, Chr_16) that consistently contained candidate genomic regions above the threshold, totaling a length of 0.8636 Mb and encompassing 61 genes. Further analysis of loci near the peak of each chromosome indicated potential association with the target trait (Table 6). Notably, no duplicated regions were found on the other chromosomes in the results from both years, underscoring the specificity of the identified candidate regions.

### 3.5. Screening Genes Related to Lodging Resistance

Previous research has established that the biosynthesis and transduction of gibberellins (GA) and indole-3-acetic acid (IAA) play an important role in plant growth and development [34]. Mutations in GA biosynthetic genes can shorten plant internode and result in plant dwarfing [35]. Initially, we identified 61 genes from four overlapping intervals. These genes were further refined based on their expression level in cell maturation zone (S) and cell elongation zone (Sh), as well as the activity of IAA promoters and inhibitors (e.g., 1–phenylpropanamide) and GA promoters and inhibitors (e.g., 5–uniconazole). Finally, we identified 12 candidate genes (Supplementary Table S2).

### 3.6. Candidate Gene Verification Using RT–qPCR Data

The stem tip serves as the primary growth point of the soybean plant, significantly influencing its growth and development processes. This region exhibits relatively high gene expression activity, and sampling from the stem tip is more accessible compared to the basal stem. In this study, six soybean varieties were selected from all varieties based on the basal stem node diameter, including three with extremely high and three with extremely low values for RT–qPCR validation: the large diameter varieties Holt (9.38 mm), Zhonghuang45 (10.73 mm), and Keshan No. 1 (8.49 mm), alongside the small diameter varieties Heihe29 (7.52 mm), Maple Ridge (7.76 mm), and Mengdou No. 9 (6.17 mm). We measured the expression levels of 12 candidate genes in these soybean varieties and found that those with smaller diameters showed higher gene expression. Furthermore, there were significant differences in gene expression between different varieties of *Glyma.02G139500*, *Glyma.03G183600*, *Glyma.03G184500* and *Glyma.16G196200*, across the varieties. Further analysis revealed that gene expression was consistently higher in the three smaller diameter cultivars, aligning with the previously observed trends (Supplementary Table S3).

### 3.7. Functional Analysis of Candidate Genes

During the analysis of the function of candidate genes within the localization interval, nine of the genes directly affected plant height and lodging resistance including *Glyma.02G138700* (regulated phosphatase activity and participated in the plant response to light stimulation), *Glyma.02G139500* (acted on the plant cell wall, leading to the rearrangement of the actin cytoskeleton of the cell wall, affecting the strength of the stem force) [36], *Glyma.03G181800* (participated in the response of the plant defense against bacteria, encoding the PRR/NLR immune receptor gene) [37], *Glyma.03G182400* (encoding members of the receptor-like cytoplasmic kinase (RLCK) subfamily VIIa, was also involved in plant defense responses against bacteria) [38], *Glyma.02G139600* and *Glyma.03G181400* (involved in plant defense responses against bacteria and plant feeding) [39,40]. The plants grew taller and were more prone to lodging under accelerators than under inhibitors. *Glyma.03G183600* belonged to the ATP-binding cassette (ABC) transporter family and was involved in light reactions and responses to plant hormones [41]. *Glyma.03G184500* participated in cell wall biosynthesis and xyloglucan metabolism, directly affecting stem breaking force [42]. *Glyma.03G184600* regulated the activity of ubiquitin protein transferase, was related to photomorphogenesis, and negatively regulated the cell biosynthesis process [43]. The other two genes indirectly affected plant lodging resistance, including the following: *Glyma.03G184200* and *Glyma.16G196200*. The former regulated FMN adenylyltransferase activity and gene expression [44], and the latter was a member of the family of heat stress transcription factors (Hsf) [45]. Furthermore, there was also a soybean-specific gene *Glyma.16G196000*, the function of which was unclear.

The candidate genes were located on three chromosomes: Chr2, Chr3, and Chr16. Among them, qPH33–3 and qPH34–3 on Chr2 had been finely mapped, they were QTLs that affect plant height and lodging resistance. The related QTLs on Chr3 and Chr16 had not been precisely mapped, and their functions were unknown, requiring further verification by genetic transformation. In addition, haplotype analysis of the above genes through public databases showed that, the haplotype of *Glyma.02G138700* in the bulk of large diameter was mainly hap_01, and the haplotype in the bulk of small diameter was mainly hap_02. The haplotype of *Glyma.03G184500* in the bulk of the large diameter was mainly hap_01, and the haplotypes in the bulk of small diameter were hap_02 and hap_04. The haplotypes of *Glyma.16G196000* in the bulk of large diameter were hap_01 and hap_02, of which hap_01 accounted for a larger proportion, and the main haplotypes in the bulk of small diameter were hap_03, hap_04, hap_12, hap_15, and hap_16, among which hap_03 accounted for a larger proportion.

## 4. Discussions

Lodging occurs primarily during the growth stage when the stems of the soybeans are too weak to support the aerial weight. Lodging makes harvesting difficult and results in significant losses in grain yield and quality. Previous studies have shown that plant height is selectively correlated with lodging resistance. During the Green Revolution, high-yield varieties of rice and wheat were developed at appropriate plant height to harvest high yield [46]. In other words, the dwarf crop varieties all grow with large diameters of basal internode so that the stems can support the above ground weight. To increase soybean yield with higher plant density than before, we need stronger soybean varieties that exhibit improved lodging resistance and yield. In dicotyledonous plants, environmental conditions that promote high yields exacerbate lodging by stimulating excessive plant height and vegetative growth [47]. Furthermore, lodging can lead to a 10% reduction in seed yield, suggesting that high-yielding germplasm may be particularly susceptible to yield losses [48]. The high density of planting increases the risk of lodging. When lodging occurs, the normal canopy structure changes due to a decrease in photosynthetic capacity and biomass production [49]. Lodging hinders the movement of water and mineral nutrients and reduces the quality and quantity of grains by assimilating them through the xylem and phloem tissues [50]. Recent studies have shown that stay-green genotypes contribute to enhanced stem lodging resistance under high plant density by reducing stem biomass reutilization [51]. Furthermore, as density increases, fierce intra-plant competition reduces the light available to coexisting plants, resulting in higher plant height and smaller stem diameter. The incidence of weeds is positively correlated with planting density, plant height, leaf sheath length, and effective tiller number, while negatively correlated with the diameter of the stem, grain number per plant, and grain weight per plant [52,53,54]. Differences in soybean stem base diameter already appear in the early growth stages of soybeans. The main genes controlling stem physiological indicators remain stable throughout the growth process and will not change significantly due to changes in developmental stages. In addition, studying the seedling stage greatly shortens the sample collection time and speeds up the experimental process. Therefore, various studies were conducted on the soybean seedling stage in this experiment. Our results give evidence that reasonably increasing the soybean planting density will increase the height of soybean plant and reduce stem diameter of the soybean plant.

Lignin, cellulose, and pectin, as highly abundant structural carbohydrates in plants, play an important role in resistance to crop lodging [55,56]. They are the main components of the cell walls and secondary cell walls [57], improving the mechanical strength and rigidity of the stems and maintaining the stability of the components of the cell wall [58,59]. Previous studies have investigated stem development and lodging resistance mechanisms in crops such as rice [60], maize [61], wheat [62], and Brassica napus [63]. Researchers have shown that genes involved in the synthesis and regulation of lignin, cellulose, and hemicellulose significantly influence lodging resistance by unraveling the molecular networks that regulate their biosynthesis and regulation [20]. The relationship between stem strength, stem rigidity, and stem composition is complex and closely intertwined [64,65]. It is generally believed that tall stems or low lignin content are unfavorable for lodging resistance. Crop lodging is greatly influenced by environmental factors, including temperature. In more detail, high temperatures significantly reduce root lodging resistance, while low temperatures and high radiation favor dry matter accumulation and increase the mechanical strength of maize stalks [56]. A research report indicated that an increase in soil temperature increases the risk of lodging in rice [66]. Subsequently, it was found that the lodging resistance in rice varieties weakened significantly with increasing temperature, but different varieties respond to temperature differently. As the average daily temperature increases in a global warming background, the total content of lignin and cellulose also increases, resulting in increased stem hardness in rice [67]. Within a certain range, an increase in temperature leads to an upward trend in the content of lignin, cellulose, and pectin in the cell walls of the plant stem, thus enhancing the reliability of this finding in the field of crop production. Compared to other yield-related traits in soybeans, there is a lack of genetic lines to resist lodging, and some germplasm exhibits unstable phenotypes due to environmental variations. Currently, little progress in soybean lodging resistance has been made. Therefore, studying changes in stem cell wall composition in response to temperature variations is of great significance to accelerate crop breeding for lodging resistance. The regulatory mechanisms affecting cell wall composition are still not fully understood, indicating the potential for further explorations from this perspective.

In recent years, the BSA–seq has applied to gene mapping successfully [68]. Previous studies have identified four QTLs (one on chromosome 7 and three on chromosome 10) related to lodging resistance [69]. In this study, we selected 499 varieties and measured the basal stem diameter for two consecutive years. After screening, we retained 85 extreme and representative natural population data. With the support of these data, we used high-throughput sequencing and BSA-seq analysis to generate ED plots. Based on the peak distribution, we identified common regions in the two datasets, including chromosomes 2, 3, 13, and 16, which comprise a total of 61 genes. Furthermore, we filtered out 12 candidate genes based on their expression levels, which were located on chromosomes 2, 3, and 16. This study provided valuable additional information on QTLs related to lodging resistance in the soybean.

**Figure 7:**
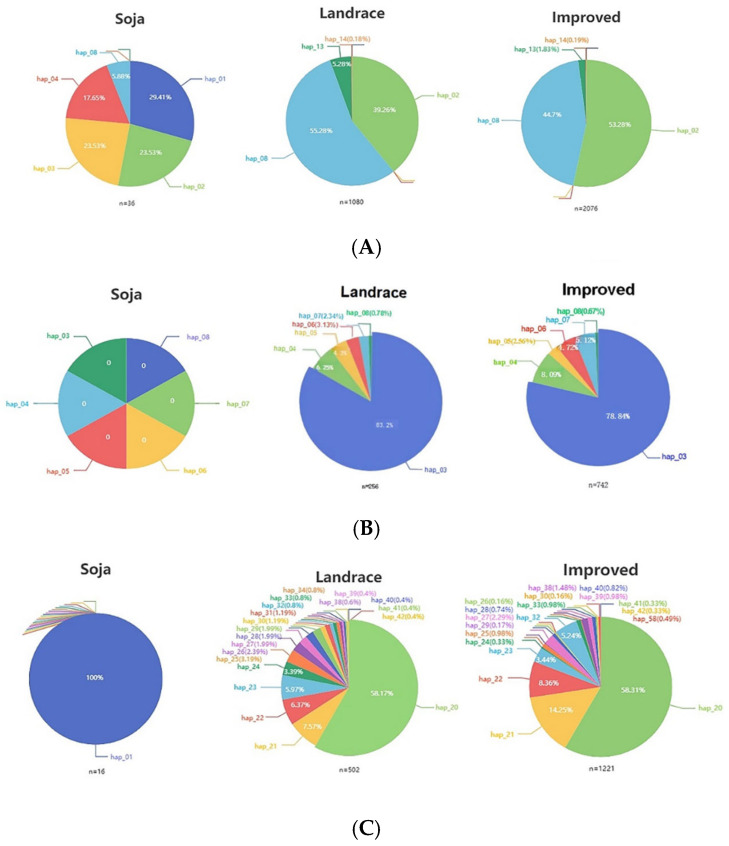
Haplotype frequency and distribution of several genes in Northeast China. Note: (**A**) *Glyma.02G138700*; (**B**) *Glyma.03G184500*; (**C**) *Glyma.16G196000*.

Subsequently, we summarized the functions of candidate genes and found that most of them were involved in plant defense responses against bacteria and responses to plant hormones, directly or indirectly regulating the lodging resistance of plants. qPH33–3 and qPH34–3 on Chr2 have been previously studied and proven, while QTLs related to plant height and lodging on Chr3 and Chr16 did not appear in previous research, indicating that they were newly discovered QTLs. Additionally, understanding the changes in gene haplotypes during the soybean domestication process is also important. In recent studies, a gene called PH13 has been identified as a determinant of plant height, providing information on the genetic basis for modern soybean breeding in high-latitude regions and offering allele variations to increase soybean yield through high-density planting [70]. The researchers performed a candidate association analysis on ZmCYP90D1 and identified favorable haplotypes in natural populations that could decrease plant height without affecting yield [71]. Given that resistance to lodging is an important agronomic trait that determines yield [72,73], we investigated whether several candidate genes have been used in the domestication and improvement processes of soybeans. Analyzing the distribution frequency of *Glyma.02G138700* haplotypes in 36 wild varieties, 1080 local varieties and 2076 improved varieties, it was found that in soybean varieties with strong resistance to lodging, the main haplotype was Hap1, while weak resistance to lodging was associated with Hap2. The proportion of Hap2 increased sequentially from wild varieties to local varieties and improved varieties. Hap8 occurred with a low frequency of only 5.88% in wild varieties but exhibited a significant increase in proportion in local and improved varieties, accounting for approximately half of all haplotypes. Similarly, we analyzed the frequency distribution of the *Glyma.03G184500* haplotypes. In soybean varieties with strong lodging resistance, the predominant haplotype was Hap1, while weak lodging resistance was associated with Hap2 and Hap4. The haplotype of this gene had not been found in wild varieties. In 256 local varieties and 742 improved varieties, Hap3 represented the majority. However, compared to local varieties, the proportion of Hap3 decreased in improved varieties. For *Glyma.16G196000*, in soybean varieties with strong lodging resistance, the main haplotype was Hap1, while weak lodging resistance was associated with Hap3. Upon comparing the haplotype frequencies, we observed that wild varieties had only Hap1, but in 502 local varieties and 1221 improved varieties, Hap1 was absent, and Hap20 accounted for more than half of the haplotypes, representing the main category of haplotypes (Figure 7). It indicates that all three genes have undergone strong artificial selection during the genetic improvement process.

## 5. Conclusions

This experiment conducted several studies on soybeans. The results showed that many factors affect soybean plant height, diameter and other indicators. Reasonable increase in soybean planting density will increase the height of soybean plants and reduce the stem diameter of soybean plants. The increase in temperature promotes the accumulation of key cell wall components in soybean seedlings, resulting in an upward trend in the content of chemical components in the cell wall of plant stems that mainly affects the strength of the stems. To identify the key QTLs that regulate soybean plant height and base stem node diameter, this study selected 499 varieties, measured the base stem diameter for 2 consecutive years, and screened 12 genes based on the expression levels in different parts and the activity of plant hormones. New QTLs related to plant height and diameter were identified on Chr3 and Chr16. At the same time, experiments have shown that the expression of stem tip genes in varieties with smaller diameters is always higher. These findings provide a theoretical basis for breeding high-yield soybean varieties with strong lodging resistance.

## Figures and Tables

**Table 1:** Primer sequences for 12 candidate genes.

Gene ID	Primer Sequence (5′-3′)	
*Glyma.02G138700*	F: AATCCCAAATGCTCTGTTCCG	R: GGTAAGGTTCTTTTCCGTCGTG
*Glyma.02G139500*	F: GACCGCAAACCCCACGATAA	R: CACCAGAAGAACCTGTTGGAGAAA
*Glyma.03G181800*	F: CAGGTCTTTGCGACATTCTACG	R: AACATCGGTGGCTTCCTCTTC
*Glyma.03G182400*	F: TGCAATTCCCAGTTTGTCCC	R: TCACTTCTGTCACCCATTCCCTA
*Glyma.02G139600*	F: CAAGCTCGTCGAGGATTTTGA	R: AACCCAGGTAACAGCACCAACTAT
*Glyma.03G183600*	F: GAGCACCGTTGTATCCCTCATT	R: CCTATCTGCTGCCTCAACCATC
*Glyma.03G184500*	F: CAAACAATGAAGCACATCCCG	R: TTGGCACATCATCCACTAGGAA
*Glyma.03G184600*	F: CAATAACGGCGAGCATCACA	R: CACATCCTTCAACGAGCATTTCAC
*Glyma.03G184200*	F: TTGGTGTTCGTTTGGCTCCT	R: CCACAATTCAGTTATGCCCTCA
*Glyma.16G196200*	F: ATCTGAGCCGTTGGATTGCC	R: CACCCTTTGATTTGTCCTGTGAC
*Glyma.16G196000*	F: AAACCAAGGCAACTCCGTCTC	R: CTGGTTGTTGGACCCATTGAA
*Glyma.03G181400*	F: GAATGGTGGAGCCGATGAGGAT	R: CCGCAATTTGGAACCGAAGA

**Table 2:** Changes in plant height and first internode stem diameter of soybeans planted at high and low densities at different stages.

Stage		Index	Plant Height/cm			Diameter/mm		
			Vn	R5	R8	Vn	R5	R8
Variety								
Heike71 (L)			14.20 ± 0.20	41.60 ± 0.63	50.10 ± 0.23	4.71 ± 0.46	6.65 ± 0.71	6.32 ± 0.17
Heike71 (H)			22.07 ± 0.03	54.00 ± 0.30	59.50 ± 0.41	4.43 ± 0.23	5.00 ± 0.02	5.42 ± 0.34
Heihe43 (L)			21.78 ± 0.88	54.58 ± 0.28	59.90 ± 0.43	5.19 ± 0.24	5.66 ± 0.06	7.22 ± 0.34
Heihe43 (H)			27.31 ± 0.30	66.50 ± 0.21	79.77 ± 0.31	4.12 ± 0.22	4.84 ± 0.37	6.16 ± 0.35
Heike88 (L)			21.80 ± 0.25	64.92 ± 0.18	65.70 ± 0.64	5.33 ± 0.27	5.37 ± 0.24	6.86 ± 0.53
Heike88 (H)			24.16 ± 0.52	73.00 ± 0.19	80.40 ± 0.40	4.12 ± 0.19	5.08 ± 0.16	5.47 ± 0.60
Heike85 (L)			20.85 ± 0.57	49.60 ± 0.13	53.50 ± 0.11	4.64 ± 0.49	5.41 ± 0.29	5.52 ± 0.49
Heike85 (H)			25.69 ± 0.27	61.83 ± 0.40	69.70 ± 0.50	4.01 ± 0.28	4.61 ± 0.29	4.71 ± 0.24

**Table 3:** Changes in hardness and resilience of soybeans planted at high and low densities in different stages.

Stage		Index	Hardness/N			Resilience/N		
			R5	R8	R8 (Test Field)	R5	R8	R8 (Test Field)
Variety								
Heike71 (L)			3887.58 ± 733	3613.83 ± 965	5226.28 ± 984	57.50 ± 0.38	57.50 ± 0.38	57.50 ± 0.38
Heike71 (H)			1333.81 ± 140	2144.71 ± 425	4678.51 ± 771	58.59 ± 1.46	58.59 ± 1.46	58.59 ± 1.46
Heihe43 (L)			3604.26 ± 80	5482.83 ± 486	11,053.16 ± 106	56.93 ± 0.43	56.93 ± 0.43	56.93 ± 0.43
Heihe43 (H)			1611.39 ± 504	2417.05 ± 359	7184.96 ± 1143	60.81 ± 0.09	60.81 ± 0.09	60.81 ± 0.09
Heike88 (L)			2070.06 ± 311	5402.55 ± 556	11,475.91 ± 219	54.21 ± 0.65	54.21 ± 0.65	54.21 ± 0.65
Heike88 (H)			1964.84 ± 597	2082.91 ± 220	5221.12 ± 1201	58.20 ± 1.23	58.20 ± 1.23	58.20 ± 1.23
Heike85 (L)			2472.86 ± 509	4424.22 ± 1564	5576.78 ± 1097	56.71 ± 0.70	56.71 ± 0.70	56.71 ± 0.70
Heike85 (H)			1020.08 ± 351	1822.26 ± 291	3572.91 ± 600	62.35 ± 0.91	62.35 ± 0.91	62.35 ± 0.91

**Table 4:** Population diameter phenotype data statistics.

Year	Max/mm	Min/mm	Mean/mm	Standard Deviation	Kutosis	Skewness
2020	12.95	2.57	7.38	1.91	0.52	0.38
2021	12.93	5.77	8.41	1.38	0.69	0.71

**Table 5:** BSA association analysis thresholds for each chromosome.

Chromosome	ED Association Analysis of Natural Populations in 2020	ED Association Analysis of Natural Populations in 2021
Chr2	0.4525	0.5091
Chr3	0.3688	0.6788
Chr13	0.5091	0.5374
Chr16	0.3538	0.4534

**Table 6:** Group overlap range between 2020 and 2021.

Chromosome	Start Location	End Location	Size/Mb
Chr2	14333783	14628947	0.2952
Chr3	41117720	41517255	0.3995
Chr13	32018780	32072589	0.0538
Chr16	35982944	36098060	0.1151

## Data Availability

Data are contained within the article and supplementary material.

## Supplementary Materials

The following supporting information can be downloaded at: https://www.mdpi.com/article/10.3390/plants13243470/s1, Table S1: 85 soybean experimental materials; Table S2: Expression levels of candidate genes in different parts of the stem and under different hormone treatments.; Table S3: Expression levels of candidate genes in soybean varieties.
