# Supplementary material for: Identification of QTLs and Key Genes Enhancing Lodging Resistance in Soybean Through Chemical and Physical Trait Analysis
Source: Plants (Basel). 2024 Dec 11;13(24):3470. doi: 10.3390/plants13243470 (PMC11728735; doi:10.3390/plants13243470)
Supplement: Supplementary file 1 [file plants-13-03470-s001.zip › plants-3323690-supplementary.pdf]

**Table S1.** 85 soybean experimental materials.

| Variety | diameter of the basal internode (mm) |
|---|---|
| Beidou19 | 10.82 |
| Beidou5 | 7.08 |
| Bell | 9.02 |
| Dengke3 | 7.26 |
| Dengke5 | 8.43 |
| Dongda1 | 7.87 |
| Dongnong33 | 8.23 |
| Dongnong37 | 7.85 |
| Dongnong46 | 12.93 |
| Dongnong50 | 7.75 |
| Dongnong59 | 9.51 |
| Dongnong61 | 7.76 |
| Dongnong68 | 11.83 |
| Fengshou11 | 7.12 |
| Fengshou23 | 6.97 |
| Fengshou27 | 9.84 |
| Fengshou9 | 6.66 |
| Haojiang11-1200 | 7.05 |
| Hefeng1 | 7.77 |
| Hefeng39 | 9.61 |
| Hefeng40 | 8.66 |
| Hefeng45 | 8.81 |
| Hefeng50 | 9.67 |
| Heidou | 7.72 |
| Heihe29 | 7.52 |
| Heihe43 | 7.71 |
| Heihe44 | 6.4 |
| Heihe45 | 8.2 |
| Heihe49 | 7.97 |
| Heihe5 | 8.92 |
| Heihe53 | 8.87 |
| Heihe53 | 9.45 |
| Heihe54 | 8.14 |
| Heijiao09-2145 | 7.93 |
| Heilongjiang41 | 8.01 |
| Heinong44 | 8.87 |
| Heinong48 | 7.71 |
| Heinong52 | 7.13 |
| Heinong531 | 7.65 |
| Heinong55 | 8.26 |
| Heinong76 | 7.72 |
| Hejiao6 | 7.7 |
| Henong91 | 7.95 |
| Holt | 9.38 |
| Jiadou19 | 10.58 |

| | |
|---|---|
| Jilin45 | 8.74 |
| Jilinxiaolidou | 10.18 |
| jinyuan55 | 9.96 |
| Jiufeng3 | 7 |
| Jiufeng4 | 7.67 |
| Jiyu608 | 7.67 |
| Jiyu609 | 7.67 |
| Kangxian4 | 8.17 |
| Kebei1 | 8.23 |
| Kenfeng18 | 10.87 |
| Kenfeng20 | 8.38 |
| Kenfeng23 | 8.74 |
| Kenfeng23 | 8.86 |
| Kennong31 | 9.86 |
| Keshan1 | 8.49 |
| Longken315 | 7.92 |
| Longpin03-311 | 9.88 |
| Maple Ridge | 7.76 |
| Mengdou13 | 8.36 |
| Mengdou31 | 6.74 |
| Mengdou9 | 6.17 |
| Nenao3 | 8.48 |
| No.12 | 8.2 |
| No.3 | 6.5 |
| No.4 | 7.16 |
| Nongda86257 | 7.94 |
| PI542042/A021 | 6.23 |
| PI548529/A030 | 6.66 |
| PI562374/A023 | 7.15 |
| PI587091/A017 | 5.77 |
| PI602060/A029 | 6.97 |
| Qi05032-4 | 8.33 |
| Suinong10 | 7.82 |
| Suinong18 | 10.91 |
| Suinong4 | 7.81 |
| Suinong76 | 8.36 |
| Yuanjinbao | 8.26 |
| Zhonghuang35 | 9.31 |
| Zhonghuang45 | 10.73 |
| Zihua4 | 9.64 |

**Table S2.** Expression levels of candidate genes in different parts of the stem and under different hormone treatments.

| Gene ID | Sh | S | GA | IAA | Uniconazole | Kynurenine |
|---|---|---|---|---|---|---|
| Glyma.02G138700 | 1.86±0.02 | 1.57±0.01 | 1.99±0.06 | 1.97±0.08 | 1.79±0.00 | 1.58±0.15 |
| Glyma.02G139500 | 1.54±0.02 | 1.27±0.01 | 1.67±0.19 | 1.73±0.20 | 1.13±0.05 | 1.03±0.21 |
| Glyma.02G139600 | 1.70±0.02 | 1.36±0.01 | 1.95±0.04 | 1.88±0.02 | 1.64±0.01 | 1.83±0.07 |
| Glyma.03G181400 | 0.89±0.02 | -0.08±0.01 | 1.44±0.07 | 1.41±0.07 | 1.33±0.02 | 1.23±0.05 |
| Glyma.03G181800 | 1.29±0.02 | 0.87±0.01 | 1.50±0.04 | 1.49±0.08 | 1.14±0.06 | 1.39±0.12 |

| | | | | | | |
|---|---|---|---|---|---|---|
| Glyma.03G182400 | 1.75±0.02 | 1.11±0.01 | 2.14±0.10 | 1.95±0.09 | 1.88±0.13 | 1.47±0.07 |
| Glyma.03G183600 | 0.40±0.02 | 0.47±0.01 | 1.09±0.08 | 0.96±0.09 | 0.83±0.06 | 0.75±0.07 |
| Glyma.03G184200 | 1.47±0.02 | 0.88±0.01 | 1.87±0.04 | 1.86±0.00 | 1.59±0.04 | 1.34±0.09 |
| Glyma.03G184500 | 1.60±0.02 | 0.67±0.01 | 0.81±0.19 | 0.38±0.02 | 0.05±0.02 | 0.63±0.09 |
| Glyma.03G184600 | 1.22±0.02 | 0.82±0.01 | 1.63±0.11 | 1.61±0.18 | 1.19±0.01 | 1.11±0.08 |
| Glyma.16G196000 | 1.41±0.02 | 1.29±0.01 | 0.00±0.00 | 2.09±1.71 | 0.00±0.00 | 2.25±0.11 |
| Glyma.16G196200 | 0.92±0.02 | 0.47±0.01 | 1.67±0.03 | 1.43±0.08 | 1.18±0.07 | 0.92±0.13 |

**Table S3.** Expression levels of candidate genes in soybean varieties.

| Gene ID | H89 | H138 | H218 | H234 | H137 | H298 |
|---|---|---|---|---|---|---|
| Glyma.02G138700 | 1.51±0.01 | 1.65±0.06 | 1.55±0.07 | 1.68±0.05 | 1.72±0.07 | 1.73±0.00 |
| Glyma.02G139500 | 1.10±0.03 | 1.42±0.01 | 1.39±0.05 | 1.35±0.04 | 1.57±0.05 | 1.39±0.04 |
| Glyma.02G139600 | 1.61±0.00 | 1.76±0.00 | 1.98±0.05 | 2.01±0.06 | 1.80±0.05 | 1.82±0.00 |
| Glyma.03G181400 | 0.97±0.08 | 1.34±0.01 | 1.27±0.01 | 1.29±0.03 | 1.24±0.00 | 1.33±0.01 |
| Glyma.03G181800 | 1.39±0.02 | 1.62±0.03 | 1.51±0.01 | 1.57±0.02 | 1.57±0.03 | 1.59±0.03 |
| Glyma.03G182400 | 1.63±0.07 | 1.91±0.07 | 1.71±0.08 | 1.80±0.00 | 1.76±0.04 | 1.77±0.01 |
| Glyma.03G183600 | 0.52±0.03 | 0.60±0.00 | 0.73±0.01 | 0.67±0.02 | 0.65±0.00 | 0.59±0.00 |
| Glyma.03G184200 | 1.22±0.10 | 1.58±0.02 | 1.54±0.02 | 1.53±0.01 | 1.50±0.01 | 1.53±0.01 |
| Glyma.03G184500 | -0.12±0.05 | 0.39±0.01 | 0.73±0.03 | 0.24±0.12 | 0.76±0.06 | 0.31±0.03 |
| Glyma.03G184600 | 0.97±0.03 | 1.30±0.00 | 1.34±0.04 | 1.38±0.00 | 1.36±0.05 | 1.24±0.02 |
| Glyma.16G196000 | 2.07±0.03 | 2.69±0.26 | 2.24±0.05 | 1.85±0.05 | 1.58±0.12 | 2.67±0.19 |
| Glyma.16G196200 | 0.65±0.15 | 1.38±0.06 | 1.52±0.00 | 1.42±0.02 | 1.18±0.02 | 1.19±0.04 |
